# Two-drug versus three-drug induction chemotherapy in pediatric acute myeloid leukemia: a randomized controlled trial

**DOI:** 10.1038/s41408-022-00726-1

**Published:** 2022-09-06

**Authors:** Venkatraman Radhakrishnan, Sameer Bakhshi, Smita Kayal, Cherian Thampy, Ankit Batra, Praveen Kumar Shenoy, Hemanth Kumar, Swaminathan Rajaraman, Shilpi Chaudhary, Reema Bisht, Biswajit Dubashi, Trivadi S. Ganesan

**Affiliations:** 1 Department of Medical Oncology, Cancer Institute (WIA) Adyar, Chennai, India; 2 Department of Medical Oncology, Dr. BRA IRCH, All India Institute of Medical Sciences, New Delhi, India; 3 Department of Medical Oncology, JIPMER, Puducherry, India; 4 Department of Biostatistics, Cancer Institute (WIA) Adyar, Chennai, India

**Keywords:** Medical research, Randomized controlled trials

## Abstract

The benefit of three-drug induction chemotherapy over a two-drug induction has not been evaluated in pediatric acute myeloid leukemia (AML). We, therefore, conducted a randomized controlled trial to ascertain the benefit of a three-drug induction regimen. Patients aged 1–18 years with newly diagnosed AML were randomized to two cycles of induction chemotherapy with daunorubicin and ara-C (DA) or two cycles of ara-C, daunorubicin, and etoposide (ADE). After induction, patients in both arms received consolidation with two cycles of high-dose ara-C. The study’s primary objective was to compare the event-free survival (EFS) between the two arms. The secondary objectives included comparing the composite complete remission (cCR) rates, overall survival (OS), and toxicities. The study randomized 149 patients, 77 in the DA and 72 in the ADE arm. The median age was 8.7 years, and 92 (62%) patients were males. The median follow-up was 50.9 months. The cCR rate in the DA and ADE arm were 82% and 79% (*p* = 0.68) after the second induction. There were 13 (17%) induction deaths in the DA arm and 12 (17%) in the ADE arm (*p* = 0.97). The 5-year EFS in the DA and ADE arm was 34.4% and 34.5%, respectively (*p* = 0.66). The 5-year OS in the DA and ADE arms was 41.4% and 42.09%, respectively (*p* = 0.74). There were no significant differences in toxicities between the regimens. There was no statistically significant difference in EFS, OS, CR, or toxicity between ADE and DA regimens in pediatric AML. The trial was registered with the Clinical Trial Registry of India (Reference number: CTRI/2014/11/005202).

## Introduction

Acute myeloid leukemia (AML) is rare in children compared to older adults [1]. Over the last few decades, there has been a significant improvement in survival in pediatric AML in high-income countries (HICs) [2]. This improvement has been achieved through better risk stratification, improved supportive care, and allogeneic hematopoietic stem cell transplantation (HSCT) [3, 4]. The outcomes of pediatric AML in low-income countries (LICs) and low and middle-income countries (LMICs) have been poor compared to HICs due to limited resources, high cost of treatment, infections, lack of access to allogeneic HSCT, delayed and moribund presentation, and treatment abandonment [5–9].

Unlike other solid and other hematological malignancies where immunotherapy and targeted therapy have changed the treatment landscape, chemotherapy remains the backbone for treating AML. Daunorubicin and ara-C (DA), also called “3 + 7” because of the 3-day daunorubicin and seven-day continuous ara-C infusion schedule, is the standard induction chemotherapy regimen for AML in adults [10]. Pediatric AML induction regimens have been heterogeneous. Most use ara-C, an anthracycline (daunorubicin, idarubicin, or mitoxantrone), and a third drug (etoposide, 6TG, or clofarabine). Randomized controlled trials (RCTs) in pediatric AML have not shown the superiority of a specific anthracycline or a specific third chemotherapy drug for induction [11–13].

Adding etoposide to the standard DA induction chemotherapy has not improved outcomes in patients with AML above 15 years [10]. There is no RCT in pediatric AML to show the superiority of a three-drug induction compared to the standard 2-drug induction. We, therefore, conducted an RCT comparing the two-drug DA regimen with the three-drug ADE regimen (DA with etoposide) in pediatric AML since ADE is the most common induction regimen used in children with AML.

## Methods

### Study design and patients

The study was an investigator-initiated, multi-center RCT conducted by the Indian Pediatric Oncology Group (InPOG) (reference number: InPOG-AML-16-01). The participating sites included: Cancer Institute (W.I.A), Chennai; All India Institute of Medical Sciences, New Delhi; and Jawaharlal Nehru Institute of Postgraduate Medical Education and Research, Puducherry. The study was approved by the Institute Ethics Committees of the participating sites and registered with the Clinical Trial Registry of India (Reference number: CTRI/2014/11/005202). Patients were recruited after obtaining written informed consent from their parents. Verbal assent was obtained from children aged 7–12 years, and written assent was obtained from children above 12 years to participate in the study.

We hypothesized that the ADE regimen would be superior to the DA regimen in improving survival in pediatric AML. The study’s primary objective was to compare the event-free survival (EFS) between the DA and ADE arms. The secondary objectives were to compare the composite complete remission (cCR) rates, toxicities, and overall survival (OS) between the two arms.

The major inclusion criteria were newly diagnosed patients with de novo AML between 1–18 years of age. The following patients were excluded from the study: children with acute promyelocytic leukemia (APML), myelodysplastic syndrome or bi-phenotypic leukemia, or Philadelphia chromosome-positive AML; serum creatinine >2 mg/dl; serum bilirubin >3 mg/dl; pregnancy; cardiac dysfunction either clinically or ejection fraction less than 50% on echocardiography; patients with hepatitis B, hepatitis C, and human immunodeficiency virus infections; previous cytotoxic chemotherapy for AML; patients with genetic disorders like downs syndrome and; the physician considered that intensive therapy was not an appropriate treatment option.

### Randomization

All consecutive patients admitted for treatment were screened for eligibility criteria. Those giving consent were randomized 1:1 to one of the pre-specified two arms (Arm A: DA or Arm B: ADE). The department of Biostatistics at the Cancer Institute assisted in the random allocation of the patients. Randomization was performed by generating random number tables through a customized computer program for the study’s proposed total number of cases. A document was then prepared to allocate all the subjects to the two arms in chronological order. The patient and the investigator were not blinded to the allocation as it was an open-label study.

### Procedures

The diagnosis of AML was confirmed on morphological examination of the bone marrow aspirate and flow cytometry. Bone marrow aspirates were sent for conventional karyotyping and polymerase chain reaction testing for t(8;21), t(15;17), t(16;16) or inversion 16, t(9;22), fms-like tyrosine kinase 3 (FLT3) gene mutation, and nucleophosmin 1 (NPM1) gene mutation.

The chemotherapy doses and schedule are provided in Table 1 [10]. The study schema is shown in Supplemental Fig. S1. Patients in Arm A received two cycles of induction with DA followed by two cycles of consolidation with high dose ara-C (HIDAC). Patients in Arm B received two cycles of induction with ADE followed by two cycles of consolidation with high dose ara-C (HIDAC). The subsequent chemotherapy cycle was initiated if the patient’s neutrophil counts recovered to 1 × 10^9^/L and platelets to 100 × 10^9^/L.

**Table 1:** Chemotherapy doses and schedule for the study regimens.

Study Arm	First Induction	Second Induction	First Consolidation	Second Consolidation
Daunorubicin and ara-C (DA)	***Daunorubicin****:*	***Daunorubicin****:*	***ara-C:***	***ara-C:***
	60 mg/m^2^ daily by IV infusion over 1 hour on days 1, 2, and 3.	60 mg/m^2^ daily by IV infusion over 1 hour on days 1, 2, and 3.	3.0 g/m^2^ 12-hourly by 4-hour IV infusion on days 1, 3 and 5.	3.0 g/m^2^ 12-hourly by 4-hour IV infusion on days 1, 3 and 5.
	***ara-C****:*	***ara-C****:*		
	100 mg/m^2^, continuous infusion days 1 to 7 inclusive.	100 mg/m^2^, continuous infusion days 1 to 7 inclusive.		
ara-C, Daunorubicin and Etoposide (ADE)	***ara-C****:*	***ara-C****:*	***ara-C:***	***ara-C:***
	100 mg/m^2^ twice daily (12 hours apart) IV push on days 1 to 10 inclusive.	100 mg/m^2^ twice daily (12 hours apart) IV push on days 1 to 8 inclusive.	3.0 g/m^2^ 12-hourly by 4-hour IV infusion on days 1, 3 and 5.	3.0 g/m^2^ 12-hourly by 4-hour IV infusion on days 1, 3 and 5.
	***Daunorubicin****:*	***Daunorubicin****:*		
	50 mg/m^2^ daily by IV infusion over 1 hour on days 1, 2 and 3.	50 mg/m^2^ daily by IV infusion over 1 hour on days 1, 2 and 3.		
	***Etoposide****:*	***Etoposide****:*		
	100 mg/m^2^ daily by 4-hour IV infusion on days 1–5 inclusive.	100 mg/m^2^ daily by 4-hour IV infusion on days 1–5 inclusive.		

*IV* Intravenous.

The bold italic values are the names of the chemotherapy drugs used.

Patients without central nervous system (CNS) disease at diagnosis received “triple” intrathecal chemotherapy with methotrexate, ara-C, and hydrocortisone, one after each of the first two courses of chemotherapy. Patients with CNS involvement received triple intrathecal chemotherapy twice a week until clearance of blasts in the cerebrospinal fluid and for a minimum of six doses, followed by triple intrathecal chemotherapy at the start of each cycle of chemotherapy.

Complete remission (CR) was defined as less than 5% blasts in the bone marrow aspirate; absence of circulating blasts and blasts with Auer rods; absence of extramedullary disease; absolute neutrophil count (ANC) ≥ 1 × 10^9^/L; and platelet count ≥100 × 10^9^/L [14]. CR with incomplete hematologic recovery (CRi) was defined as CR criteria except for ANC less than 1 × 10^9^/L or platelet count less than 100×10^9^/L [14]. cCR was defined as CR + CRi. A bone marrow aspirate was performed to assess the remission status after 21 days and beyond after starting the first induction, provided the patient had a hematological recovery defined as an absolute neutrophil count (ANC) of more than 1.5 × 10^9^/L and platelets of more than 75 × 10^9^/L. If the bone marrow was hypoplastic and assessment of remission status was not possible, a repeat bone marrow aspiration was performed after an additional 7–10 days to assess the remission status. A bone marrow aspirate was performed on day 42 if the patient did not achieve hematological recovery. Patients in whom the bone marrow remission status was not interpretable after two bone marrows proceeded to receive the second induction based on the investigator’s evaluation of the clinical status. The bone marrow was re-assessed 21 days and beyond after starting the second induction if the patient did not achieve CR after the first induction or if the bone marrow was not evaluable after the first induction. The criteria for bone marrow assessment after the first induction was followed for the second. The disease was considered refractory if the patient did not achieve CR after the second induction, and the patient was withdrawn from the trial. The treatment of patients with refractory disease was based on the treating center’s discretion. Minimal residual disease (MRD) assessment was not performed in the study as it was not available for AML at the treating centers until 2019.

Patients with t(8;21), inv 16 or t(16;16), and NPM1 mutation without FLT-3 internal tandem duplication (ITD) were considered favorable-risk. Patients with FLT-3 ITD, complex cytogenetics (more than three abnormalities), t(6;9), t(3;3), −5, del 5q or −7 were classified as adverse risk. Patients who did not meet either low or high-risk criteria were defined as intermediate risk [14]. Karyotyping, PCR, and morphology were not reviewed centrally.

Allogenic HSCT was considered in patients not in CR after the second induction (refractory disease) and in patients with intermediate or adverse risk, irrespective of the remission status. The decision to proceed with the transplant was based on the availability of donors, finances, physician, and parent preferences. Relapse was defined as bone marrow blasts ≥5%; reappearance of blasts in the blood; or development of extramedullary disease. Treatment-related toxicities were graded according to the National Cancer Institute (NCI) Common Terminology Criteria for Adverse Events (CTCAE) v4.0 [15]. The World Health Organization (WHO) AnthroPlus software was used to calculate the patient’s nutritional status at baseline [16]. Patients under five years with a weight for age less than −2 standard deviation (SD) and those five years and above with body mass index (BMI) for age less than −2 SD were considered undernourished. Obesity was defined as weight for age more than +2 SD (under five years) or BMI for age more than +2 SD (five years and above).

### Protocol modification

The study protocol was modified in June 2017 after it was expanded as a multi-centric study. The primary endpoint was changed from CR rate to EFS based on the inputs of the investigators. This increased the sample size from 186 to 188.

### Statistical analysis

The sample was calculated based on a 20% difference in EFS with 80% power and a 5% chance level. Ninety-four patients were needed to be recruited in each arm to improve EFS in the DA arm from 35% to 55% in the ADE arm. The EFS of 35% in the DA arm was calculated based on our retrospective pediatric AML data [17]. The improvement of EFS to 55% was based on the outcomes from pediatric AML trials using ADE induction. The Ethics Committee closed the study in May 2020 after the accrual of 149 patients instead of the planned recruitment of 188 patients due to the slow accrual rate over six years, observations of the data safety board on the futility of continuing the study as the outcomes of the study arms were similar and the COVID19 pandemic. Descriptive statistics were used to analyze all the patient’s demographic and clinical characteristics. Comparison between categorical variables was made by chi-square test. The independent student t-Test was used to compare means. An event in the trial was defined as disease relapse, progression (refractory), or death due to any cause. The EFS was calculated from the date of the diagnosis to the date of the event. The OS was calculated from the date of diagnosis to the date of death or last follow-up. The Kaplan-Meier survival analysis was used to calculate the EFS and OS, and results were reported with 95% confidence intervals (CI). The log-rank test was used to compare variables for survival analysis. Multivariate survival analysis was performed using the Cox regression model. All tests were two-sided, and a significance level of 0.05 was used. All statistical analyses were performed on SPSS (IBM, Chicago, version 17).

An intention-to-treat analysis was performed. Treatment abandonment was defined as the discontinuation of the planned treatment by the study participant. A study participant was considered lost to follow-up if the participant did not visit the hospital for a review after completing the study treatment or could not be contacted telephonically for more than a year since the last visit or contact.

## Results

**Fig. 1:**
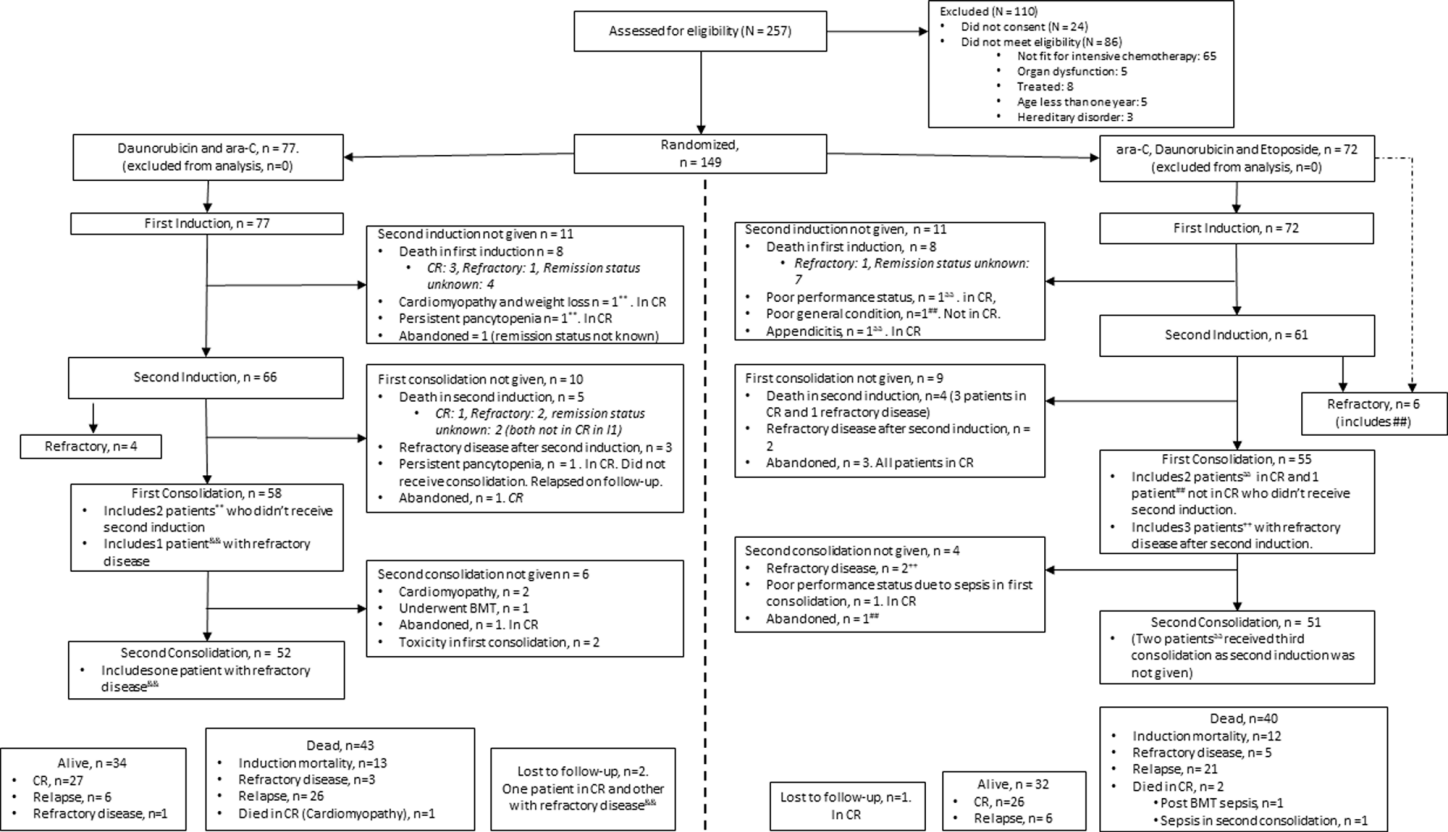
Study consort diagram.

Between 1^st^ June 2014 and 14^th^ November 2019, 149 patients were enrolled and randomized. Seventy-seven patients were randomized to the DA and 72 to the ADE arm. The consort diagram is shown (Fig. 1). The median age was 8.7 years (range: 1–18 years), and 92 (62%) patients were males. Thirty-three (43%) patients in the DA arm and twenty-five (35%) in the ADE arm were undernourished at diagnosis. The baseline characteristics of patients were comparable between the two arms (Table 2).

**Table 2:** Demographic, laboratory, and clinical characteristics of the patients in the study.

	DA (*n* = 77) (%)	ADE (*n* = 72)	*p* value
Trial Site			
1	49 (63.6)	46 (63.8)	0.98
2	22 (28.5)	20 (27.7)	
3	6 (7.7)	6 (8.3)	
Age in years			
Median (range)	8.12 (1.25–18)	8.95 (1–17.4)	0.77
Age (years)			
1–4.99	25 (32.4)	25 (34.7)	0.88
5–9.99	19 (24.6)	14 (19.4)	
10–14.99	10 (12.9)	9 (12.5)	
15–18	23 (29.8)	24 (33.3)	
Sex			
Male	49 (63.6)	43 (59.7)	0.62
Female	28 (36.3)	29 (40.2)	
WBC count (mm^3^)			
<10,000	28 (36.3)	24 (33.3)	0.07
10,000–49,999	35 (45.4)	22 (30.5)	
50,000–99,999	7 (9)	12 (16.6)	
>100,000	7 (9)	14 (19.4)	
Platelet count (mm^3^)			
<10,000	0	1 (1.3)	0.77
10,000–49,999	43 (55.8)	39 (54.1)	
50,000–99,999	25 (32.4)	26 (36.1)	
100,000–149,999	5 (6.4)	5 (6.9)	
>150,000	4 (5.1)	1 (1.3)	
Hemoglobin (gms%)			
<7.99	48 (62.3)	48 (66.6)	0.81
8–9.99	22 (28.5)	18 (25)	
10–11.99	5 (6.4)	3 (4.1)	
> 12	2 (2.5)	3 (4.1)	
Subtype			
M0	1 (1.2)	1 (1.3)	0.76
M1	1 (1.2)	5 (6.9)	
M2	27 (35)	26 (36.1)	
M4/M5	20 (25.9)	16 (22.2)	
M6	1 (1.2)	1 (1.3)	
M7	3 (3.8)	2 (2.7)	
Not available	24 (3.1)	21 (29.1)	
Nutritional status			
Undernourished	33 (42.8)	25 (34.7)	0.59
Normal	43 (55.8)	46 (63.8)	
Obese	1 (1.2)	1 (1.3)	
Albumin			
Mean, Standard Deviation	3.56, 0.9	3.71, 0.87	0.31
<3.5 gms/dL	29 (37.6)	23 (31.9)	
Tumor Lysis Syndrome	5 (6.4)	6 (8.3)	0.66
Cytogenetic Risk			
Favorable	27 (35)	23 (31.9)	0.52
Intermediate	31 (40.2)	30 (41.6)	
Adverse	18 (23.3)	15 (20.8)	
Not Available	1 (1.2)	4 (5.5)	
NPM			
Mutated	3 (3.8)	9 (12.5)	0.02
Unmutated	72 (93.5)	56 (77.7)	
Not Available	2 (2.5)	7 (9.7)	
FLT3			
Tyrosine Kinase Domain	0	0	0.18
Internal Tandem Duplication	9 (11.6)	8 (11.1)	
Not mutated	66 (85.7)	57 (79.1)	
Not available	2 (2.5)	7 (9.7)	
Fever at diagnosis			
Yes	24 (31.1)	14 (19.4)	0.10
Extramedullary disease (Chloroma)	14 (18.1)	7 (9.7)	0.13
CNS involvement	3 (3.8)	4 (5.5)	0.63

*DA* Daunorubicin and ARAC, *ADE* ARAC, Daunorubicin, and Etoposide, *TLS* Tumor Lysis Syndrome, *NPM* Nucleophosphamin, *FLT3* FMS Like Tyrosine Kinase 3, *CNS* Central Nervous System.

### Response to induction and induction mortality

After the first induction, the DA and ADE arm’s cCR rate was 65% and 68% (*p* = 0.68), respectively. It was 82% and 79% (*p* = 0.68) after the second induction (Table 3). Two patients had a non-evaluable first bone marrow examination after the first induction and five patients had a non-evaluable first bone marrow examination after the second induction. All the above patients had evaluable bone marrow at the second attempt except one patient after the first induction. This patient achieved CR after the second induction. The median time for performing the first bone marrow post-first induction was 23 days in the DA arm and 24.5 days in the ADE arm. The median time for performing the first-bone marrow post-induction second induction was 23 days in the DA arm and 23 days in the ADE arm (Table 3).

**Table 3:** Comparison of outcomes between the DA and ADE arms.

	DA (*n* = 77) (%)	ADE (*n* = 72) (%)	*p* value
Composite complete remission after I1 (CR + CRi)			
Yes^^^	50 (65)	49 (68)	0.68
CR	45 (90)	47 (96)	
CRi	5 (10)	2 (4)	
No	22 (28.5)	15 (20.8)	
I1 bone marrow evaluation not done			
Died	4	7	0.89
Abandonment	1	0	
Not evaluable	0	1	
Composite complete remission after I2			
(CR + CRi)	50 + 13 = 63 (82)	49 + 8^^^^=57 (79)	0.68
Yes (I1 + I2)^@^			
CR	12 (92)	6 (75)	
CRi	1 (8)	2 (25)	
No	6	6	
I2 bone marrow evaluation not done			
Died	3	1	0.76
Abandonment	0	1	
Not evaluable	0	0	
Time to I1 bone marrow evaluation (days)			
Median	23	24.5	0.15
Mean, SD	24.21 (4.24)	25.27 (4.37)	
Time to I2 bone marrow evaluation (days)			
Median	23	23	0.87
Mean, SD	23.06 (4.05)	23.29 (4.03)	
Induction Mortality			
Total	13 (16.8)	12 (16.6)	0.97
I1	8 (10.3)	8 (11.1)	
I2	5 (6.4)	4 (5.5)	
Cause for induction mortality			
Sepsis	11 (14.2)	9 (12.5)	0.74
Intracranial hemorrhage	1	1	
Pulmonary hemorrhage	0	2	
Gastrointestinal hemorrhage	1	0	
Consolidation mortality	0	1	0.96
Cause of consolidation mortality			
Febrile neutropenia	0	1	0.9
Duration of I1 (days)***			
Median	26	26	0.46^+^
Mean, SD	23.01, 10.33	21.72, 10.86	
Duration of I2 (days)***			
Median	22	22	0.93^+^
Mean, SD	20.33, 8.73	20.44, 6.9	
Dose reduction			
Total	14 (18.1)	5 (6.9)	0.03
I1	2	4	
I2	1	0	
First consolidation	4	0	
Second consolidation	7	1	
Positive blood culture in I1 (patients)			
Total	14 (18.1)	8 (11.1)	0.22
Gram-positive/MDR	3/2	4/2	
Gram-negative/ MDR	11/2	4/2	
Positive blood culture in I2 (patients)			
Total	8 (10.3)	5 (6.9)	0.45
Gram-positive/MDR	2/1	1/0	
Gram-negative/MDR	6/3	4/3	
Number of days of antibiotics in I1			
Mean, SD	16.79, 5.62	16.01, 6.47	0.78^+^
Number of days of antibiotics in I2			
Mean, SD	11.61, 5.32	9.57, 6.18	0.05^+^
Antibiotics use in I1^#^ (number of patients)			
None	0	0	0.95
First line	20	16	
Second line	17	15	
Third line	40	40	
Antibiotics use in I2^#^ (number of patients)			
None	2	6	0.08
First line	30	22	
Second line	8	15	
Third line	26	18	
I1 PRBC transfusions			
Mean, SD	4.32, 2.94	4.42, 3.72	0.85^+^
I1 platelet transfusions			
Mean, SD	17.72, 15.20	16.1, 14.2	0.5^+^
I2 PRBC transfusions			
Mean, SD	1.88, 1.67	2, 2	0.37^+^
I2 platelet transfusions			
Mean, SD	8.54, 9.18	6.25, 7.21	0.13^+^
Relapses (morphological)	32	27	0.61
Second-line chemotherapy at relapse	11 (14.2)	9 (12.5)	0.74
Allogenic HSCT			
Total	10 (12.9)	4 (5.5)	0.12
First complete morphological remission (CR1)	4	2	
Second complete morphological remission (CR2)	6	2	
HSCT outcome			
Alive	5	2	0.28
CR1 transplant	2	0	
CR2 transplant	3	2	
Treatment abandonment	3 (3.8)	4 (5.5)	0.63

*I1* First induction, *I2* Second induction, *DA* Daunorubicin and ara-C, *ADE* ara-C, Daunorubicin, and Etoposide, *MDR* Multi-Drug Resistant, *PRBC* Packed Red Blood Cells, *HSCT* Hematopoietic Stem Cell Transplantation, ^+^t-Test. ^^^Includes patients who had postmortem bone marrow aspiration.

^@^Patients in complete remission (CR) or CR with incomplete recovery (CRi) in I1 did not undergo bone marrow after I2 unless there was a suspicion of disease relapse. CR rates in I2 include patients who achieved CR in I1.

^#^ First line (Cefaperazone sulbactam with teicoplanin or amikacin, cefepime), Second line, (Meropenem, imipenem), Third line (Colistin, tigecycline).

^^^^Includes patients with not evaluable bone marrow in first induction.

*** Reply: The duration of I1 was from the day of the start of I1 to the start of I2. The duration of I2 was defined from the day of the start of I2 to the start of the first HIDAC. If patients did not start I2 or consolidation due to mortality or treatment abandonment, the date of death or abandonment was used to calculate the duration.

There were 13 (17%) induction deaths in the DA arm (8 in the first and 5 in the second induction) and 12 (17%) in the ADE arm (8 in the first and 4 in the second induction) (*p* = 0.69). Eleven out of 13 (84.6%) induction deaths in the DA arm and nine out of the 12 (75%) in ADE were due to sepsis (Table 3). Nine out of 13 patients (69.2%) who died during induction in the DA arm and 7 out of 12 patients (58.3%) in the ADE arm were undernourished. There was no induction mortality in obese patients. The induction mortality details are provided in Supplemental Table S1.

### Supportive Care During Induction

The mean duration of hospitalization during the first induction in the DA and ADE arm was 23.01 and 21.72 days (*p* = 0.46). The mean duration of hospitalization during the second induction in the DA and ADE arm was 20.3 and 20.4 days (*p* = 0.93). There were no significant differences between the DA and ADE arms during the two cycles of induction for blood product support, days of antibiotic use, lines of antibiotic use, therapeutic anti-fungal use, and blood culture positivity (Table 3).

### Refractory disease

Nine patients had a refractory disease after completing two cycles of induction, four in DA and five in the ADE arm (Supplemental Table S2). The remission status of seven out of 13 patients who died during induction was unknown as post-mortem bone marrow studies could not be performed (Supplemental Table S1). One patient with refractory disease after the first cycle of induction received HIDAC consolidation omitting the second induction due to poor performance status. This patient abandoned the treatment after the first HIDAC. All except one patient with the refractory disease died at the last follow-up, whose survival status could not be ascertained. Only one patient with refractory disease underwent allogeneic HSCT after achieving CR with salvage chemotherapy. This patient died post-HSCT due to sepsis and encephalitis.

### Consolidation

Fifty-eight out of 77 (75.3%) patients in the DA arm and 55 out of 72 (76.3%) patients in the ADE arm received the first HIDAC consolidation. Fifty-two out of 77 (67.5%) patients in the DA arm and 51 out of 72 (70.8%) patients in the ADE arm received the second HIDAC consolidation (Fig. 1). Two patients in the DA arm (one patient developed cardiomyopathy and the other persistent pancytopenia) and three patients in the ADE arm (one patient developed appendicitis and the other two had poor performance status due to septic shock) did not receive the second induction. They were planned for three HIDAC consolidations. One patient with the refractory disease in the DA arm received two cycles of HIDAC. This patient was later lost to follow-up. Three patients with refractory disease in the ADE arm after the second induction received HIDAC consolidation; two received one cycle of HIDAC consolidation, and one patient received two cycles of HIDAC followed by allogeneic HSCT. There were no deaths during consolidation in the DA arm. One patient in the ADE arm died due to febrile neutropenia and sepsis after receiving the second HIDAC cycle.

### Toxicities

Grade 3–5 toxicities across all chemotherapy cycles are reported in Table 4. There were no significant differences in toxicities between the study arms. Cardiomyopathy and mucositis/diarrhea were the most common toxicities in the study arms.

**Table 4:** Comparison of grade 3–5 toxicities between the two study arms.

	DA (*n* = 77)	ADE (*n* = 72)	*p* value
Cardiac	5 (6.5%)	6 (8.3%)	0.66
Renal	2 (2.6%)	5 (6.9%)	0.21
Hepatic	4 (5.2%)	5 (6.9%)	0.65
Neurological	3 (3.9%)	0	0.66
Mucositis/Diarrhea	5 (6.5%)	6 (8.3%)	0.66
Pulmonary	1 (1.2%)	3 (4.1%)	0.27
I1 Febrile neutropenia	77 (100%)	72 (100%)	0.96
I2 Febrile neutropenia	64/66 (97%)	55/61 (90%)	0.11

*DA* Daunorubicin and ARAC, *ADE* ARAC, Daunorubicin and Etoposide, *I1* First induction, *I2* Second induction.

Two deaths post induction were treatment-related. One patient in the DA arm developed cardiomyopathy after the first HIDAC cycle. This patient did not receive the second HIDAC cycle and died due to cardiomyopathy four months after the first HIDAC cycle. One patient in the ADE arm died due to febrile neutropenia and sepsis after receiving the second HIDAC cycle.

### Relapses

Fifty-nine (39.5%) patients relapsed after achieving CR, 32/77 (41.5%) in DA and 27/72 (37.5%) in the ADE arm (*p* = 0.61). The mean and median time to relapse in the whole cohort was 17.55 and 11.07 months (SD: 17.19, range: 3.47–91 months). The mean and median time to relapse in the DA arm was 17.08 and 10.16 months (SD:18.58, range: 3.6-91 months). The mean and median time to relapse in the ADE arm was 18.1 and 14.13 months (SD: 15.71, range: 3.47-73.6 months). There was no significant difference in the mean time to relapse between the study arms (*p* = 0.82). All patients relapsed in the bone marrow; there were no extramedullary relapses. Twenty-one out of 59 (35.5%) patients received salvage chemotherapy (12 in the DA arm and 9 in the ADE arm), among whom eight underwent allogeneic HSCT (6 in the DA and 2 in the ADE arm). At the last follow-up, nine patients were alive and in CR including five patients who underwent allogeneic HSCT (3 in DA and 2 in the ADE arm).

### Allogenic HSCT

Fourteen patients received allogeneic HSCT, ten in DA (four in first remission and six in second) and four (two in first remission and two in second) in the ADE arm. Seven out of 14 patients are alive post HSCT, all in CR (Table 3).

One hundred patients were eligible for allogeneic HSCT in first remission, this included 61 patients with intermediate risk, 33 with adverse risk, 5 with risk not classified, and one with favorable risk and refractory disease. However, only 6 patients underwent HSCT in the first CR. The reasons for not performing allogeneic HSCT in the first CR in 94 eligible patients are described in Supplemental Table S3.

### Survival outcome

The median duration of follow-up was 50.9 months (95% CI: 40.3–63.8 months). Three patients abandoned treatment in the DA arm and four in the ADE arm. The details of treatment abandonment have been provided in Supplemental Table S4. Three patients were lost to follow-up after the planned treatment, two in the DA arm and one in the ADE arm. One patient in the DA arm in CR was lost to follow-up after 34 months and seven days of completing treatment; the second patient in the DA arm had refractory disease after two cycles of induction and was lost to follow-up after 16 months and 17 days of completing the second HIDAC. The patient in the ADE arm was in CR and was lost to follow-up after 31 months.

**Fig. 2:**
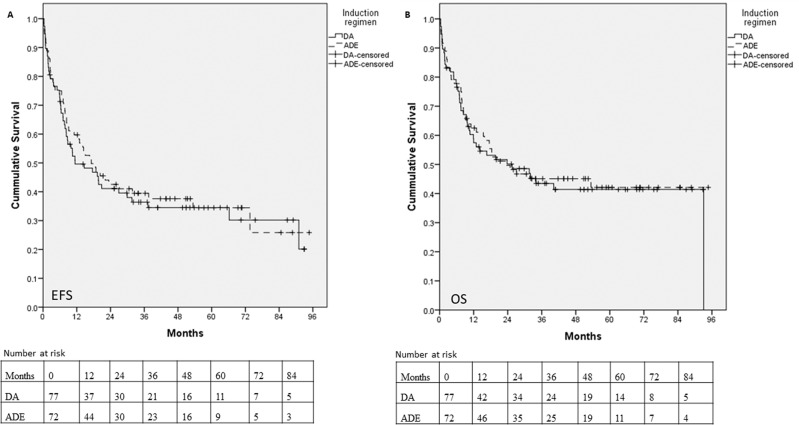
Kaplan-Meier survival curves. **A** comparing Event Free Survival (EFS) between Daunorubicin and ARAC (DA) arm and ARAC, Daunorubicin, and Etoposide (ADE) arm (*p* = 0.66), and **B** comparing Overall Survival (OS) between DA arm and ADE arm (*p* = 0.74).

The 5-year EFS in the DA and ADE arm was 34.4% (95% CI: 23.6–45.6) and 34.5% (95% CI: 22.7–46.4), respectively (*p* = 0.66) (Fig. 2A). The 5-year OS in the DA and ADE arm was 41.4% (95% CI: 29.5–52.8) and 42.09% (95% CI: 29.7–53.9), respectively (*p* = 0.74) (Fig. 2B).

The median EFS and OS in the whole cohort were 16.57 (95% CI: 10.14–23) and 23.77 months (95% CI: 11.41–36.13). The median EFS in the DA arm was 11.37 months (95% CI: 1.49–21.24), and the ADE arm was 17.17 months (95% CI: 9.07–25.26) (*p* = 0.66). The median OS in the DA arm was 25.53 months (95% CI: 6.22–44.8), and in the ADE arm was 23.33 months (95% CI: 0–54.51) (*p* = 0.74). There was no significant difference in EFS and OS for eligible patients who underwent HSCT in the first remission versus eligible patients who did not (Supplemental Fig. S2A and B) and patients who underwent HSCT in the first or second remission versus those who did not (Supplemental Fig. S2C and D).

Univariate and multivariate analyses for factors predicting the EFS and OS in the total cohort, DA arm, and ADE arm have been provided in Supplemental Tables S5 to S12. On multivariate analysis for EFS in the ADE arm, CR after the first induction (*p* < 0.001), age below ten years (*p* < 0.001), and WBC count less than 50,000/cumm (*p* = 0.011) were associated with better EFS. On multivariate analysis for OS in the ADE arm, CR after the first induction (*p* = 0.007), age below ten years (*p* < 0.001), and trial site (*p* = 0.016) were associated with better OS. None of the variables predicted EFS and OS in multivariate analysis in the DA arm. For the whole cohort, inferior EFS and OS were associated with not achieving CR after the first induction (*p* = 0.001 and 0.002, respectively), and the trial site (*p* = 0.027 and 0.012, respectively), and undernutrition at diagnosis predicted inferior OS (*p* = 0.014).

## Discussion

Collaborative groups in the United Kingdom, the United States of America, and Germany have used a three-drug induction regimen in their ongoing research protocols or recently published studies (Supplemental Table S13). The primary objective of our study was to compare the EFS between a 2-drug DA induction regimen with a 3-drug ADE induction regimen. We wanted to evaluate if adding a third drug to 2-drug induction improved survival outcomes and if it was associated with more toxicities and increased supportive care requirements. Knowing this is important in resource-constrained settings in LICs and LMICs where treatment of pediatric AML is challenging due to the high prevalence of gram-negative multi-drug resistant (MDR) bacterial infections, financial constraints, and restricted supportive care.

The spectrum of bacterial infections in LICs and LMICs is different from HICs [18]. Gram-negative and MDR infections are more common among patients with cancer in LICs and LMICs [18, 19]. In contrast, gram-positive infections are more common in HICs [20]. A high proportion of patients with acute leukemia in LMICs are colonized with gram-negative MDR bacteria in stool at admission, indicating that these were community-acquired [21, 22]. MDR gram-negative bacterial infections are associated with a high incidence of mortality [23, 24]. Twenty out of 25 (80%) induction mortality in our study was due to sepsis (Table 3). Twenty-five out of 35 (71%) positive blood cultures during the induction period in the study were due to gram-negative bacteria, among whom 10 (40%) had MDR bacteria (Table 3). Six out of 10 (60%) patients with MDR gram-negative bacteremia died. Addressing gram-negative infections and MDR bacteria is vital to improving survival in pediatric AML in LICs and LMICs.

Our study did not find a significant difference in EFS between the DA and ADE arms (5-year EFS: 34.4% and 34.5%, respectively, *p* = 0.66). There were no significant differences between the ADE and DA arms regarding toxicities and supportive care requirements. However, since we did not achieve our planned accrual, our study is not adequately powered to confirm our findings.

Options for treatment after relapse in pediatric AML are limited in developing countries due to the high cost of salvage chemotherapy, allogeneic HSCT, and limited donor availability. Though HSCT in the first CR was advised for intermediate and high-risk patients, only 6% underwent allogeneic HSCT. The participating centers performed only matched sibling donor HSCT until 2018, limiting the number of patients receiving HSCT. Other factors related to meager rates of HSCT were the high cost of salvage chemotherapy and HSCT, protracted treatment course and follow-up prohibiting long-term commitment from the family/caregiver, family’s apprehension of the risk to the donor, and overall transplant outcomes, besides other socioeconomic issues. Only 21 out of 59 (35.5%) patients who relapsed in the study received chemotherapy at relapse. The high cost of salvage chemotherapy at relapse and HSCT was the most common reason why most patients at relapse did not undergo HSCT in our study. Access to HSCT is limited to most patients with AML in LMICs and is not unique to our study. The participating centers in the trial are routinely performing haploidentical HSCT and matched unrelated donor transplants since 2018; this has increased the number of patients eligible for HSCT [25]. The inclusion of HSCT under state insurance has also reduced the cost, improving access to the procedure.

The induction mortality, the CR rates, and the 5-year EFS and OS reported from our study are inferior to the results from trials from HICs (Supplemental Table S13). Ninety percent of children with cancers are treated in LICs and LMICs, and our results reflect the reality for most children with AML [26]. A recent systematic review of literature on outcomes of pediatric AML in LMICs included 27 studies, 26 retrospective, and one prospective observational study [7]. The 5-year EFS varied from 24% to 63%, and the 5-year OS from 10% to 72% [7]. The authors concluded that outcomes of pediatric AML in LMICs are substantially inferior compared to HICs [7]. There is heterogeneity in the outcomes reported from LMICs. The inferior outcomes in LMICs are due to high abandonment rates, early deaths, treatment-related mortality, and limited treatment options after relapse [7].

Thirty-nine percent of the study patients were undernourished at diagnosis, and 1.3% were obese. On univariate analysis, undernutrition at diagnosis was associated with inferior EFS and OS in the whole cohort and the ADE arm but not in the DA arm (Supplemental Tables S5 and S6). On multivariate analysis, undernutrition at diagnosis was one of the factors associated with inferior OS (Supplemental Table S8). In contrast, in the NOPHO-AML 2004 study, only 5% of patients were undernourished [27]. In the NOPHO-AML 2004 study, there was a trend toward better overall survival in obese children (23% of patients) above ten years [27]. In the Children’s Cancer Group 2961 study, 10.9% were underweight, and 14.8% were overweight. Survival was inferior, and treatment-related mortalities were higher in underweight and overweight patients than in normal-weight patients [28]. Aggressive nutritional support and tailoring cytotoxic treatment to the patient’s nutritional status might improve the overall outcomes in pediatric AML in LICs and LMICs.

Sixty-five patients screened for enrollment in the study were not eligible as they were not fit for intensive chemotherapy. This subgroup equals 44% of patients enrolled in the trial. Many patients in LICs and LMICs present late and in moribund condition. Most are malnourished and have ongoing infections or organ dysfunction requiring intensive care. Hence, they cannot be given intensive chemotherapy due to the high risk of mortality from chemotherapy [29].

Patients who achieved CR after the first induction in our study had significantly better survival than those who did not. This was observed in the whole cohort and those who received the ADE regimen. Whether a second induction is necessary after achieving CR with the first induction needs to be explored. Omitting second induction and replacing it with HIDAC consolidation in pediatric AML can reduce the risk of cardiac toxicity and second malignancies due to a reduction in anthracycline and etoposide cumulative dose. Patients who do not achieve CR after the first induction should be considered for intensive chemotherapy followed by allogeneic HSCT, as our study shows that they have inferior outcomes even if they achieve CR after the second course of induction, especially in the ADE arm.

Based on our sub-group analysis, an etoposide-based three-drug induction might be more effective in patients less than ten years of age, with favorable cytogenetics, those with an extramedullary disease, and those who are not malnourished (Supplemental Tables S5, S6, S9 and S10).

We used etoposide as the third drug in our intervention arm as the ADE regimen is the most common three-drug induction used in pediatric AML trials (Supplemental Table 13) and routine clinical practice. Studies in adult AML have shown increased CR rates and survival with the addition of fludarabine or cladribine to induction chemotherapy regimens [10, 30, 31]. The AML-08 trial compared clofarabine and ara-C induction with ADE in pediatric AML [32]. The study showed increased MRD negative rates with clofarabine-based induction, however, this did not translate to improvements in EFS or OS [32]. The addition of fludarabine or cladribine or clofarabine and omitting etoposide in pediatric AML induction needs further evaluation.

The limitations of our study include the inability to achieve the desired sample size, the absence of MRD-based risk-stratification, and limited access to HSCT. The dose and schedule of daunorubicin and ara-C were different between the DA and ADE arms. We used a dose and schedule of DA and ADE regimens used in routine clinical practice and reported in the literature. However, our study is the first RCT on pediatric AML from LICs and LMICs. The study also challenges the dogma of three-drug induction chemotherapy in pediatric AML.

To conclude, no statistically significant difference in EFS, OS, CR, or toxicity between ADE and DA regimens in pediatric patients with AML was observed in our study. Future RCTs are needed to find the optimal number of induction chemotherapy drugs and cycles in pediatric AML.

## Supplementary information

Supplementary Tables

Supplementary Figure 1

Supplementary Figure 2

## Data Availability

The data that support the findings of this study are available on request from the corresponding author. The data are not publicly available due to privacy or ethical restrictions.
