# Correction: Two-drug versus three-drug induction chemotherapy in pediatric acute myeloid leukemia: a randomized controlled trial

**DOI:** 10.1038/s41408-022-00735-0

**Published:** 2022-09-27

**Authors:** Venkatraman Radhakrishnan, Sameer Bakhshi, Smita Kayal, Cherian Thampy, Ankit Batra, Praveen Kumar Shenoy, Hemanth Kumar, Swaminathan Rajaraman, Shilpi Chaudhary, Reema Bisht, Biswajit Dubashi, Trivadi S. Ganesan

**Affiliations:** 1 Department of Medical Oncology, Cancer Institute (WIA) Adyar, Chennai, India; 2 Department of Medical Oncology, Dr. BRA IRCH, All India Institute of Medical Sciences, New Delhi, India; 3 Department of Medical Oncology, JIPMER, Puducherry, India; 4 Department of Biostatistics, Cancer Institute (WIA) Adyar, Chennai, India

**Keywords:** Medical research, Randomized controlled trials

Correction to: *Blood Cancer Journal* 10.1038/s41408-022-00726-1, published online 06 September 2022

In this article the affiliation details for Shilpi Chaudhary and Reema Bisht were incorrectly assigned as ‘3’ but should have been ‘2’.

