# Supplementary material for: Two-drug versus three-drug induction chemotherapy in pediatric acute myeloid leukemia: a randomized controlled trial
Source: Blood Cancer J. 2022 Sep 6;12(9):131. doi: 10.1038/s41408-022-00726-1 (PMC9444698; doi:10.1038/s41408-022-00726-1)
Supplement: Supplementary file 1 — Supplementary Tables [file 41408_2022_726_MOESM1_ESM.docx]

**Supplemental Table S1**. Details of induction mortality

| **Patient**  **number** | **Induction phase** | **Day of mortality** | **Nutritional status** | **Remission status (Bone marrow)** | **Cause of mortality** |
| --- | --- | --- | --- | --- | --- |
| DA |  |  |  |  |  |
| 1 | First | 22 | Normal | CR | Sepsis |
| 2 | First | 13 | Normal | CR | Sepsis |
| 3 | First | 24 | Undernourished | CR | Gastrointestinal bleeding |
| 4 | First | 27 | Undernourished | Not in CR | Refractory disease with sepsis |
| 5 | First | 16 | Undernourished | BMA not done | Sepsis |
| 6 | First | 20 | Undernourished | BMA not done | Intracranial bleed |
| 7 | First | 6 | Undernourished | BMA not done | Sepsis |
| 8 | First | 3 | Undernourished | BMA not done | Neutropenic colitis and sepsis |
| 9 | Second | 15 | Normal | I1 BMA: Not in CR  I2 BMA: Not in CR | Sepsis, refractory disease |
| 10 | Second | 18 | Undernourished | I1 BMA: Not in CR  I2 BMA: Not in CR | Sepsis, refractory disease |
| 11 | Second | 16 | Undernourished | I1 BMA: CR | Sepsis |
| 12 | Second | 15 | Normal | I1 BMA: Not in CR  I2 BMA: Not done | Sepsis |
| 13 | Second | 7 | Undernourished | I1 BMA: Not in CR  I2 BMA: Not done | Fungal pneumonia |
| ADE |  |  |  |  |  |
| 1 | First | 1 | Normal | BMA not done | Pulmonary hemorrhage |
| 2 | First | 4 | Normal | BMA not done | Pneumonia and Pulmonary hemorrhage |
| 3 | First | 9 | Undernourished | Not in CR | Sepsis/DIC |
| 4 | First | 31 | Normal | BMA not done | Sepsis/ Pulmonary hemorrhage |
| 5 | First | 13 | Undernourished | BMA not done | Sepsis |
| 6 | First | 7 | Normal | BMA not done | Sepsis |
| 7 | First | 4 | Undernourished | BMA not done | Intracranial bleed |
| 8 | First | 7 | Undernourished | BMA not done | Neutropenic colitis and sepsis |
| 9 | Second | 26 | Undernourished | I1 BMA: CR  I2 BMA: Not done | Sepsis |
| 10 | Second | 16 | Undernourished | I1 BMA: Not in CR  I2 BMA: Not in CR | Sepsis/DIC |
| 11 | Second | 26 | Undernourished | I1 BMA: Not in CR  I2 BMA: CR | Sepsis |
| 12 | Second | 24 | Normal | I1 BMA: CR  I2 BMA: Not done | Pulmonary hemorrhage |

Abbreviations. DA: Daunorubicin and ara-C; ADE: ara-C, Daunorubicin and Etoposide; I1: First Induction. I2: Second Induction. BMA: Bone Marrow Aspiration; CR: Complete Remission. DIC: Disseminated Intravascular Coagulation

**Supplemental Table S2**. The outcomes of patients with refractory disease after second induction (excludes patients who died with refractory disease in first or second induction)

| **Patient number** | **Study arm** | **Treatment** | **Outcome** |
| --- | --- | --- | --- |
| 1 | DA | OMC | Died |
| 2 | DA | Two cycles of HIDAC | Alive, lost to follow-up |
| 3 | DA | BSC | Died |
| 4 | DA | OMC | Died |
| 5 | ADE | OMC | Died |
| 6 | ADE | OMC | Died |
| 7 | ADE | Two cycles of HIDAC followed by salvage chemotherapy and allogeneic HSCT | Died post HSCT due to sepsis. In CR. |
| 8 | ADE | Received one cycle HIDAC followed by OMC | Died |
| 9 | ADE | Received one cycle HIDAC | Died |
| 10 | ADE | Was not in CR after the first induction. The second induction was omitted, and the patient received the first HIDAC consolidation. Abandoned treatment after the first HIDAC consolidation. | Died |

Abbreviations. DA: Daunorubicin and ara-C; ADE: ara-C, Daunorubicin, and Etoposide OMC: Oral Metronomic Chemotherapy; BSC: Best Supportive Care; HIDAC: High Dose ara-C; I2: Second Induction. HSCT: Hematopoietic Stem Cell Transplantation. CR: Complete Remission.

Note: The OMC schedule included oral cyclophosphamide 25-50 mg/m^2^/day and oral etoposide 25-50 mg/m^2^/day given continuously till progression or unacceptable toxicity.

**Supplemental Table S3**. Reasons for not performing allogeneic hematopoietic stem cell transplantation (HSCT) in first complete remission.

| **Reason** | **Number (%)** |
| --- | --- |
| **First Remission** | **N=94/100 (94%)** |
| No sibling match | 30 (32) |
| Induction mortality | 20 (21) |
| Financial | 19 (20) |
| Family not willing to transplant | 12 (13) |
| Refractory disease with poor performance status and not fit for HSCT | 8 (9) |
| Lost to follow-up | 2 (2) |
| Treatment toxicity (cardiac) | 2 (2) |
| Disease progression while waiting for transplant | 1 (1) |
| **Second Remission/Relapse** | **N= 51/59^*^ (86%)** |
| Financial (salvage chemotherapy and HSCT) | 22 (43) |
| No sibling match | 10 (20) |
| Poor performance status/organ dysfunction | 8 (15) |
| Family not willing to transplant | 6 (12) |
| Refractory disease/Died during salvage chemotherapy | 4 (8) |
| Relapsed awaiting transplant | 1 (2) |

Note: Six patients underwent allogeneic HSCT in the first remission and eight in the second remission.

^*^ Includes 3 patients who were transplanted in the first remission.

**Supplemental Table S4.** Treatment abandonment details

| **Patient**  **number** | **Phase of abandonment** | **Disease status** | **Reason for abandonment** | **Patient status^*^** |
| --- | --- | --- | --- | --- |
| **DA arm** | | | | |
| 1 | First induction | Not known as post-induction bone marrow was not done | The family did not want to continue treatment. | Died at home |
| 2 | Second induction | In remission | The family wanted to try alternative medicine. | Not known |
| 3 | After the first consolidation | In remission after I2 | Did not take the second consolidation. Reason not known | Not known |
| **ADE arm** | | | | |
| 1 | First consolidation | In remission | Did not take the first consolidation. The family did not want to continue treatment. | Relapsed and died. |
| 2 | First consolidation | In remission | Did not take the first consolidation. The family did not want to continue treatment. | Relapsed and died. |
| 3 | First consolidation | In remission | Did not take the first consolidation. The family did not want to continue treatment. | Relapsed and died. |
| 4 | Second consolidation | Not in remission | Was not in remission after the first induction. The second induction was omitted because of poor performance status, and the patient was started on HIDAC consolidation. The family refused further treatment. | Died at home. |

Abbreviations: DA: Daunorubicin and ara-C; ADE: ara-C, Daunorubicin and Etoposide; HIDAC: High Dose ara-C. ^*^ Confirmed telephonically.

**Supplemental Table S5**. Univariate analysis of parameters for Event Free Survival

| **Characteristic** | **Total Cohort** | **5-year EFS % (95% CI)** | **P value** | **DA arm** | **5-year EFS % (95% CI)** | **P value** | **ADE Arm** | **5-year EFS % (95% CI)** | **P value** |
| --- | --- | --- | --- | --- | --- | --- | --- | --- | --- |
| EFS | 149 | 34.5  (26.3-42.7) |  | 77 | 34.4  (23.6-45.6) |  | 72 | 34.5  (22.7-46.4) |  |
| Induction Regimen  DA  ADE | 77  72 | 34.4  (23.6-45.6)  34.5  (22.7-46.4) | 0.66 | - | - | - | - | - | - |
| Age  <10 years  ≥ 10 years | 82  67 | 37.4  (26.7-48)  30.9  (19.1-43.4) | 0.99 | 44  33 | 24.2  (12.3-38.3)  48.6  (30.2-64.7) | **0.02** | 38  34 | 51.8  (34.7-66.4)  13.4  (3.2-30.8) | **0.01** |
| Nutrition  Normal or obese  Undernourished | 91  58 | 42  (31.3-52.2)  21.7  (10.8-35.1) | **0.004** | 44  33 | 40.1  (24.8-54.9)  25.6  (11.7-42) | 0.06 | 47  25 | 43.6  (28.9-57.3)  13.3  (13.2-39) | **0.02** |
| WBC  <50,000/cumm  ≥50,000/cumm | 109  40 | 33.09  (23.4-43)  37.1  (22.4-51.8) | 0.87 | 63  14 | 30  (18.4-42.5)  50  (22.8-72.2) | 0.35 | 46  26 | 36.8  (21.3-52.3)  29.6  (13.4-47.8) | 0.46 |
| Infection at diagnosis  No  Yes | 111  38 | 36.5  (26.6-46.4)  27.1  (14.1-41.9) | 0.18 | 53  24 | 36  (22.6-49.6)  30.7  (13.7-49.6) | 0.64 | 58  14 | 37.3  (23.6-51)  21.4  (5.2-44.7) | 0.12 |
| Risk stratification  Favorable  Intermediate  Adverse | 50  61  33 | 47.9  (32.5-61.7)  23.2  (12.9-35.2)  33  (17.8-49) | **0.006** | 27  31  18 | 44  (24.1-62.3)  28.6  (13.5-45.7)  33.3  (13.6-54.5) | 0.3 | 23  30  15 | 52.4  (28.8-71.4)  18.6  (6.8-34.9)  33.3  (12.1-56.4) | **0.008** |
| Extramedullary disease  Absent  Present | 128  21 | 32.5  (24-41.4)  46.7  (24.6-66.1) | 0.3 | 63  14 | 36.5  (24.2-48.7)  25.7  (6.6-50.6) | 0.52 | 65  7 | 28.9  (17.5-41.3)  85.1  (33.4-97.8) | **0.02** |
| CR after the first induction  Yes  No | 99  37 | 44.7  (33.8-54.9)  19.9  (8.7-34.1) | **<0.001** | 50  22 | 42.6  (28.1-56.3)  24.6  (9-44.1) | **0.03** | 49  15 | 46.4  (30.3-61.1)  13.3  (2.1-34.5) | **<0.001** |
| Time to achieve CR  First induction  Second induction | 99  24 | 44.7  (33.8-54.9)  30.8  (13.8-49.8) | 0.054 | 50  16 | 42.6  (28.1-56.3)  34.09  (12.5-57.2) | 0.41 | 49  8 | 46.4  (30.3-61.1)  25  (3.7-55.8) | **0.047** |
| Trial site  1  2  3 | 95  42  12 | 38.6  (28.5-48.6)  32.2  (18.1-47.3)  14.2  (0.9-43.9) | **0.035** | 49  22  6 | 40.1  (26.3-53.5)  27.4  (8.7-50.2)  0 | **0.007** | 46  20  6 | 37.02  (22.6-51.4)  35  (15.6-55.1)  25  (1.2-64.5) | 0.49 |

Abbreviations. EFS: Event Free Survival. CI: Confidence Interval; CR: Complete Remission; DA: Daunorubicin and ara-C; ADE: ara-C, Daunorubicin, and Etoposide

**Supplemental Table S6**. Univariate analysis of parameters for Overall Survival

| **Characteristic** | **Total Cohort** | **5-year OS % (95% CI)** | **P value** | **DA arm** | **5-year OS % (95% CI)** | **P value** | **ADE Arm** | **5-year OS % (95% CI)** | **P value** |
| --- | --- | --- | --- | --- | --- | --- | --- | --- | --- |
| Overall Survival | 149 | 41.7  (33.1-50) | - | 77 | 41.4  (29.5-52.8) | - | 72 | 42.09  (29.7-53.9) | - |
| Induction Regimen  DA  ADE | 77  72 | 41.4  (29.5-52.8)  42.09  (29.7-53.9) | 0.74 | - | - | - | - | - | - |
| Sex  Male  Female | 92  57 | 38.8  (28.3-49.1)  46.6  (32.4-59.6) | 0.32 | 49  28 | 33.7  (20.1-47.8)  55.1  (34.3-71.8) | 0.09 | 43  29 | 44.8  (29.3-59.2)  39.2  (20.9-57.1) | 0.72 |
| Age  Age  <10 years  ≥ 10 years | 82  67 | 44.6  (33.3-55.2)  37.9  (25-50.7) | 0.8 | 44  33 | 31.9  (17.9-46.8)  54.3  (35.1-70.1) | 0.1 | 38  34 | 57.8  (40.7-71.6)  22  (7.8-40.8) | **0.02** |
| Nutrition  Normal or obese  Undernourished | 91  58 | 51.6  (40.6-61.5)  23.8  (11.7-38.2) | **0.001** | 44  33 | 47.8  (31.9-62.1)  31.4  (15-49.3) | 0.13 | 47  25 | 55.05  (39.7-67.9)  12.71  (12.6-38.1) | **0.003** |
| WBC  <50,000/cumm  ≥50,000/cumm | 109  40 | 39.8  (29.5-49.9)  45  (29.3-59.4) | 0.98 | 63  14 | 38.5  (25.4-51.5)  50  (22.8-72.2) | 0.75 | 46  26 | 41.2  (25-56.6)  42.3  (23.4-60) | 0.72 |
| Infection at diagnosis  No  Yes | 111  38 | 32.3  (18-47.4)  44.5  (34.1-54.4) | 0.068 | 53  24 | 44.2  (29.3-58.1)  35  (16.8-54) | 0.31 | 58  14 | 44.9  (30.4-58.3)  28.5  (8.8-52.3) | 0.12 |
| Risk stratification^*^  Favorable  Intermediate  Adverse | 50  61  33 | 56.7  (40.9-69.8)  30.5  (18.7-43.1)  39.1  (22.7-55.1) | **0.018** | 27  31  18 | 51  (29.7-68.9)  36.2  (18.6-54.1)  38.8  (17.4-59.9) | 0.36 | 23  30  15 | 64.06  (40.6-80.2)  25.7  (11.6-42.4)  40  (16.4-62.7) | **0.028** |
| Extramedullary disease  Absent  Present | 128  21 | 39.2  (30.2-48.1)  58.3  (33.1-76.9) | 0.15 | 63  14 | 41.3  (28.5-53.6)  39  (11.2-66.7) | 0.82 | 65  7 | 37.4  (25-49.8)  85.7  (33.4-97.8) | **0.048** |
| CR after first induction^+^  Yes  No | 99  37 | 53.8  (42.2-63.7)  24.4  (11.8-39.5) | **<0.001** | 50  22 | 49.6  (34-63.3)  33  (14.2-53.2) | 0.06 | 49  15 | 57.9  (41.1-71.4)  13.3  (2.1-34.5) | **<0.001** |
| Time to achieve CR  First induction  Second induction | 99  24 | 53.8  (42.5-63.7)  35.2  (16.9-54.2) | **0.037** | 50  16 | 49.6  (34-63.3)  40.9  (17.09-63.6) | 0.38 | 49  8 | 57.9  (41.1-71.4)  25  (3.7-55.8) | **0.03** |
| Trial site  1  2  3 | 95  42  12 | 48.2  (37.5-58.09)  32.4  (16.2-49.8)  14.06  (0.9-43.5) | **0.011** | 49  22  6 | 48.3  (33.6-61.5)  28.9  (6.7-56.6)  0 | **0.005** | 46  20  6 | 48.2  (32.6-62.3)  35  (15.6-55.1)  25  (1.2-64.5) | 0.22 |

Abbreviations. OS: Overall Survival. CI: Confidence Interval; CR: Complete Remission; DA: Daunorubicin and ara-C; ADE: ara-C, Daunorubicin, and Etoposide. * Excludes cases where risk stratification was not available. ^+^ Excludes cases where bone marrow aspiration was not done due to induction mortality, bone marrow not evaluable, or patient abandoned treatment.

**Supplemental Table S7**. Multivariate analysis of parameters for Event Free Survival for the whole cohort

| **Effect** | **Variable** | **Hazard Ratio (95% CI)** | **P-Value** |
| --- | --- | --- | --- |
| CR after the first induction | Yes vs. No | 0.42 (0.26-0.69) | **0.001** |
| Nutritional Status | Normal or Obese vs. Undernourished | 0.64 (0.39-1.06) | 0.086 |
| Sex | Male vs. Female | 0.86 (0.53-1.38) | 0.54 |
| Age | <10 years vs. ≥10 years | 0.81(0.49-1.34) | 0.42 |
| WBC | <50,000/cumm vs ≥50,000/cumm | 1.05 (0.63-1.77) | 0.83 |
| Infection at diagnosis | No vs. Yes | 0.71 (0.41-1.23) | 0.22 |
| Risk stratification | Overall  Favorable vs. Intermediate  Intermediate vs. Adverse | 0.64 (0.33-1.25)  1.26 (0.69-2.32) | 0.07  0.19  0.44 |
| Extramedullary disease | Absent vs. Present | 1.01 (0.49-2.1) | 0.96 |
| Induction Regimen | DA vs. ADE | 0.95 (0.60-1.5) | 0.85 |
| Trial Site | Overall  Site 1 vs. 2  Site 2 vs. 3 | 0.37 (0.17-0.82)  0.62 (0.24-1.55) | **0.027**  0.014  0.30 |

Abbreviations. CI: Confidence Interval; CR: Complete Remission; DA: Daunorubicin and ara-C; ADE: ara-C, Daunorubicin, and Etoposide

**Supplemental Table S8**. Multivariate analysis of parameters for Overall Survival for the whole cohort.

| **Effect** | **Variable** | **Hazard Ratio (95% CI)** | **P-Value** |
| --- | --- | --- | --- |
| CR after the first induction | Yes vs. No | 0.43 (0.25-0.73) | **0.002** |
| Nutritional Status | Normal or Obese vs. Undernourished | 0.51 (0.30-0.87) | **0.014** |
| Sex | Male vs. Female | 0.98 (0.58-1.68) | 0.96 |
| Age | < 10 years vs. ≥10 years | 0.71 (0.41-1.23) | 0.22 |
| WBC | <50,000/cumm vs ≥50,000/cumm | 0.94 (0.53-1.66) | 0.84 |
| Infection at diagnosis | No vs. Yes | 0.60 (0.33-1.07) | 0.08 |
| Risk stratification | Overall  Favorable vs. Intermediate  Intermediate vs. Adverse | 0.59 (0.28-1.24)  0.96 (0.50-1.84) | 0.27  0.16  0.92 |
| Extramedullary disease | Absent vs. Present | 1.25 (0.53-2.93) | 0.60 |
| Induction Regimen | DA vs. ADE | 0.96 (0.58-1.59) | 0.89 |
| Trial Site | Overall  Site 1 vs. 2  Site 2 vs. 3 | 0.35 (0.16-0.8)  0.73 (0.27-1.94) | 0.012  0.012  0.53 |

Abbreviations. CI: Confidence Interval; CR: Complete Remission; DA: Daunorubicin and ara-C; ADE: ara-C, Daunorubicin, and Etoposide

**Supplemental Table S9**. Multivariate analysis of parameters for Event Free Survival in ADE arm

| **Effect** | **Variable** | **Hazard Ratio (95% CI)** | **P-Value** |
| --- | --- | --- | --- |
| CR after the first induction | Yes vs. No | 0.20 (0.07-0.53) | **0.001** |
| Nutritional Status | Normal or Obese vs. Undernourished | 0.99 (0.45-2.18) | 0.99 |
| Sex | Male vs. Female | 0.72 (0.36-1.47) | 0.37 |
| Age | <10 years vs. ≥10 years | 0.14 (0.05-0.37) | **<0.001** |
| WBC | <50,000/cumm vs ≥50,000/cumm | 0.35 (0.16-0.78) | **0.011** |
| Infection at diagnosis | No vs. Yes | 0.49 (0.16-1.45) | 0.20 |
| Risk stratification | Overall  Favorable vs. Intermediate  Intermediate vs. Adverse | 1.27 (0.43-3.73)  2.46 (1.01-5.95) | 0.09  0.65  0.046 |
| Extramedullary disease | Absent vs. Present | 4.73 (0.52-42.6) | 0.16 |
| Trial Site | Overall  Site 1 vs. 2  Site 2 vs. 3 | 0.52 (0.14-1.84)  1.91 (0.37-9.88) | 0.055  0.31  0.43 |

Abbreviations. CI: Confidence Interval; CR: Complete Remission; ADE: ara-C, Daunorubicin, and Etoposide.

**Supplemental Table S10**. Multivariate analysis of parameters for Overall Survival in ADE arm

| **Effect** | **Variable** | **Hazard Ratio (95% CI)** | **P-Value** |
| --- | --- | --- | --- |
| CR after the first induction | Yes vs. No | 0.25 (0.09-0.69) | **0.007** |
| Nutritional Status | Normal or Obese vs. Undernourished | 0.50 (0.22-1.10) | 0.087 |
| Sex | Male vs. Female | 0.53 (0.23-1.1) | 0.11 |
| Age | <10 years vs. ≥10 years | 0.13 (0.04-0.41) | **<0.001** |
| WBC | <50,000/cumm vs ≥50,000/cumm | 0.48 (0.20-1.16) | 0.1 |
| Infection at diagnosis | No vs. Yes | 0.45 (0.14-1.43) | 0.17 |
| Risk stratification | Overall  Favorable vs. Intermediate  Intermediate vs. Adverse | 1.00 (0.29-3.3)  1.54 (0.59-4) | 0.56  0.99  0.37 |
| Extramedullary disease | Absent vs. Present | 3.23 (0.33-31.2) | 0.31 |
| Trial Site | Overall  Site 1 vs. 2  Site 2 vs. 3 | 0.47 (0.12-1.73)  2.86 (0.51-15.8) | **0.016**  0.25  0.22 |

Abbreviations. CI: Confidence Interval; CR: Complete Remission; ADE: ara-C, Daunorubicin, and Etoposide.

**Supplemental Table S11**. Multivariate analysis of parameters for EFS in DA arm

| **Effect** | **Variable** | **Hazard Ratio (95% CI)** | **P-Value** |
| --- | --- | --- | --- |
| CR after the first induction | Yes vs. No | 0.52 (0.26-1.02) | 0.06 |
| Nutritional Status | Normal or Obese vs. Undernourished | 0.56 (0.26-1.20) | 0.13 |
| Sex | Male vs. Female | 1.12 (0.58-2.16) | 0.72 |
| Age | <10 years vs. ≥10 years | 1.91 (0.93-3.91) | 0.07 |
| WBC | <50,000/cumm vs ≥50,000/cumm | 1.11 (0.44-2.77) | 0.82 |
| Infection at diagnosis | No vs. Yes | 0.85 (0.41-1.76) | 0.66 |
| Risk stratification | Overall  Favorable vs. Intermediate  Intermediate vs. Adverse | 0.49 (0.19-1.22)  0.71 (0.27-1.89) | 0.29  0.12  0.50 |
| Extramedullary disease | Absent vs. Present | 0.72 (0.3-1.71) | 0.46 |
| Trial Site | Overall  Site 1 vs. 2  Site 2 vs. 3 | 0.33 (0.1-1.04)  0.30 (0.07-1.24) | 0.15  0.06  0.09 |

Abbreviations. CI: Confidence Interval; CR: Complete Remission; DA: Daunorubicin and ara-C.

**Supplemental Table S12**. Multivariate analysis of parameters for Overall Survival in DA arm

| **Effect** | **Variable** | **Hazard Ratio (95% CI)** | **P-Value** |
| --- | --- | --- | --- |
| CR after the first induction | Yes vs. No | 0.60 (0.29-1.25) | 0.17 |
| Nutritional Status | Normal or Obese vs. Undernourished | 0.60 (0.26-1.38) | 0.23 |
| Sex | Male vs. Female | 1.79 (0.82-3.93) | 0.14 |
| Age | <10 years vs. ≥10 years | 1.91 (0.86-4.24) | 0.1 |
| WBC | <50,000/cumm vs ≥50,000/cumm | 0.85 (0.33-2.14) | 0.73 |
| Infection at diagnosis | No vs. Yes | 0.68 (0.31-1.48) | 0.33 |
| Risk stratification | Overall  Favorable vs. Intermediate  Intermediate vs. Adverse | 0.47 (0.17-1.26)  0.56 (0.20-1.55) | 0.31  0.13  0.26 |
| Extramedullary disease | Absent vs. Present | 1.08 (0.38-3.06) | 0.88 |
| Trial Site | Overall  Site 1 vs. 2  Site 2 vs. 3 | 0.35 (0.16-0.8)  0.73 (0.27-1.94) | 0.1  0.034  0.09 |

Abbreviations. CI: Confidence Interval; CR: Complete Remission; DA: Daunorubicin and ara-C.

**Supplemental Table S13**. Comparison of major clinical trials for pediatric AML.

| **Trial (reference)** | **Enrollment period** | **Country** | **Age included** | **Total patients** | **Total cycles of induction** | **Number of drugs in induction** | **Drugs used in induction** | **Consolidation cycles** | **CR rate %** | **Induction Mortality** | **Cytogenetic risk** | **Survival outcome** | **Primary objective** |
| --- | --- | --- | --- | --- | --- | --- | --- | --- | --- | --- | --- | --- | --- |
| MRC 10^1^ | 1988-1995 | UK, Ireland, and New Zealand | <16 | 364 | 2 | 3 | DAT vs ADE | 2 | 83 | 4.9% | Favorable: 25%.  Unfavorable: 9% | 7-y EFS: 48%  7-y OS: 56% | Comparison of DAT vs. ADE induction. |
| BFM 93^2^ | 1993-1998 | Germany, Austria, and Switzerland | <17 | 471 | 1 | 3 | ADE Vs AIE | 2 cycles consolidation. 2 cycles intensification. Maintenance 1 year. | 82.2 | 7.4% | Favorable: 28.6%. | 5-y EFS: 50%  5-y OS: 58% | Role of high dose cytarabine and mitoxantrone (HAM) consolidation. |
| MRC AML12^3^ | 1995-2002 | UK, Netherlands, and New Zealand | <16 | 529 | 2 | 3 | ADE vs MAE | 3 | 92 | 1.78 | Favorable: 28.4%.  Intermediate: 55.3%  Adverse: 14% | 10-y EFS: 54%  10-y OS: 64% | Comparison of ADE vs. MAE induction and Four vs. Five course treatment. |
| CCG2961^4^ | 1996-2002 | USA | < 21 | 901 | 2 | 5 | IdaDCTER | 1-2 cycles. BMT. IL-2. | 88 | 10.9%  (after phase 2) | Favorable: 25%.*  Normal: 70%.  Unfavorable: 5%. | 5-y EFS: 52%  5-y OS: 42% | To study role of idarubicin, fludarabine and IL-2. |
| AML-BFM 98^5^ | 1998-2003 | Germany, Austria, Switzerland and Czech Republic | < 18 | 473 | 2 | 3 | AIE. HAM | 3 cycles followed by maintenance. | 88.4 | 3.2% | Favorable: 28.4%. | 5-y EFS: 49%  5-y OS: 62% | Randomization between shorter consolidation and G-CSF use. |
| AML 99^6^ | 2000-2002 | Japan | <18 | 240 | 1.  Two cycles if M3 BM | 3 | Age < 2yr or WBC < 100,000/μL: MAE  Age ≥ 2 yr and WBC ≥ 100,000/μL: AIE | 5-6 | 94.6 | 1.4% | Favorable: 52.5%.* | 5-y EFS: 61.6%  5-y OS: 75.6% | Use of dose-dense intensive chemotherapy regimen and an appropriate risk stratification system. |
| St Jude AML02^7^ | 2002-2008 | USA | ≤ 21 | 216 | 2 | 3-4 | ADE. Addition of GO in induction 2. | 3 | 94 | 0.9% | Favorable: 26.3%. | 3-y EFS: 63%  3-y OS: 71% | Randomization between high dose and low dose ara-C in induction 1. Addition of GO in induction 2 for MRD positive. |
| MRC 15^8^ | 2002-2009 | UK | 0-73 years | 104 (less than 14 years) | 2 | 3 | ADE vs FLAG-IDA | 2 vs 3 | NA | NA | NA | NA | Comparison of DA vs. ADE vs. FLAG-IDA. Less than 14 years compared ADE vs. FLAG-IDA and 2 versus 3 HIDAC consolidations. |
| AIEOP 2002^9^ | 2002-2011 | Italy | <18 years | 482 | 2 | 3 | AIE | 3 | 87% | 2.69% | Favorable: 25.5%.* | 8-y EFS: 55%  8-y OS: 68% | Risk-adapated treatment. Auto or allo HSCT in high-risk |
| COG AAML03P1^10^ | 2003-2005 | USA | ≤ 21 | 350 | 2 | 3-4 | ADE with GO (first course). | 3 | 87 | 2.6% | Favorable: 25%.  Normal: 71%  High: 4% | 3-y EFS: 53%  3-y OS: 66% | Safety of adding 2 doses of GO to intensive chemotherapy during remission induction and postremission intensification. |
| NOPHO-AML 2004^11^ | 2004-2009 | Denmark, Finland, Iceland,  Norway, Sweden, and Hong Kong | ≤ 18 | 151 | 2 | 4 and 2 | Induction 1: AIET Induction 2: AM | 4 | 92 | 6.08% | Favorable: 30.8%. | 3-y EFS: 57%  3-y OS: 69% | Post consolidation randomization to GO or no  further therapy. |
| BFM 2004^12^ | 2004-2010 | Germany, Austria, Switzerland, and the Czech Republic | <18 | 521 | 2 | 3 | First cycle AIE vs. ADE.  Second cycle  AI vs. HAM | 2 cycles consolidation. 1-2 cycles intensification. Maintenance. | 88.2% | 2.3% | Favorable: 21.1%.*  Unfavorable: 5% | 5-y EFS:55%  5-y OS: 74%% | Liposomal daunorubicin vs idarubicin. |
| AAML0531^13^ | 2006-2010 | USA | <29 | 1022 | 2 | 3 or 4 | ADE with or without GO | 2 | 86.7% | 2.34% | Favorable: 24%.  Intermediate: 59.3%  Unfavorable: 16.5% | 3-y EFS: 53.1% in GO arm vs 46.9% in non-GO arm  3-y OS: 69.4% in GO arm vs 65.4% in non-GO arm | To study the effect of adding GO to standard chemotherapy. |
| AML08^14^ | 2008-2017 | USA | <22 | 285 | 2 | 2 or 3 | ADE vs Clofarabine + ARA-C | 3 | 92.3% | 1.1% | Favourable: 20.9%* | 3-y EFS: 52.9% Clofarabine arm vs.52.4% ADE arm.  3-y OS:  74.8% Clofarabine arm vs. 64.6% ADE arm. | Addition of Clofarabine into the first induction to reduce exposure to daunorubicin and etoposide. |
| AAML103^15^ | 2011-2016 | USA | <29.5 | 1097 | 2 | 3 or 4 | ADE with or without bortezomib | 2 | 84% | 2.3% | Favorable: 46%.  Unfavorable: 5% | 3-y EFS: 45.9%  3-y OS: 65.4% | Addition of bortezomib to standard chemotherapy. |
| InPOG-AML-16-01 | 2014-2019 | India | 1-18 | 149 | 2 | 2 vs. 3 | DA vs. ADE | 2 | 80.5 | 16.7% | Favorable: 33.5%.  Intermediate: 40.9%  Unfavorable: 22.1% | 5-y EFS: 34.5%  5-y OS: 41.4% | Comparing ADE vs DA induction. |

Abbreviations. AML: Acute Myeloid Leukemia; DA: Daunorubicin and ara-C; ADE: ara-C, Daunorubicin, and Etoposide; DAT: Daunorubicin, ara-C and 6-Thioguanine; AIE: ara-C, Idarubicin, Etoposide; MAE: Mitoxantrone, ara-C and Etoposide; IdaDCTER: idarubicin, ara-C, etoposide, thioguanine, dexamethasone; IL-2: Interleukin-2; HAM: high- ara-C and mitoxantrone; GO: Gemtuzumab Ozogamicin ; FLAG-IDA: Fludarabine, Cytarabine, G-CSF, Idarubicin; AIET: ara-C, Idarubicin, etoposide, and 6-thioguanine); AM: ara-C and Mitoxantrone; AI: ara-C and Idarubicin.

BFM: Berlin, Frankfurt Munster; MRC: Medical Research Council; COG: Children's Oncology Group; NOPHO: Nordic Society of Paediatric Haematology and Oncology; CCG: Children's Cancer Group; AIEOP: Associazione Italiana Ematologia Oncologia Pediatrica; InPOG: Indian Pediatric Oncology Group.

EFS: Event Free Survival; OS: Overall Survival; BMT: Bone Marrow Transplant. G-CSF: Granulocyte Colony Stimulating Factor. NA: Not Available. HSCT: Hematopoietic Stem Cell Transplantation.

* t(8;21) and inv 16.

**References**

1. Hann IM, Stevens RF, Goldstone AH, et al. Randomized comparison of DAT versus ADE as induction chemotherapy in children and younger adults with acute myeloid leukemia. Results of the Medical Research Council’s 10th AML trial (MRC AML10). Adult and Childhood Leukaemia Working Parties of the Medical Research Council. *Blood*. 1997;89(7):2311-2318.

2. Creutzig U, Ritter J, Zimmermann M, et al. Idarubicin improves blast cell clearance during induction therapy in children with AML: results of study AML-BFM 93. *Leukemia*. 2001;15(3):348-354. doi:10.1038/sj.leu.2402046

3. Gibson BES, Webb DKH, Howman AJ, et al. Results of a randomized trial in children with Acute Myeloid Leukaemia: Medical Research Council AML12 trial. *Br J Haematol*. 2011;155(3):366-376. doi:10.1111/j.1365-2141.2011.08851.x

4. Lange BJ, Smith FO, Feusner J, et al. Outcomes in CCG-2961, a Children’s Oncology Group Phase 3 Trial for untreated pediatric acute myeloid leukemia: a report from the Children’s Oncology Group. *Blood*. 2008;111(3):1044-1053. doi:10.1182/blood-2007-04-084293

5. Creutzig U, Zimmermann M, Lehrnbecher T, et al. Less Toxicity by Optimizing Chemotherapy, but Not by Addition of Granulocyte Colony-Stimulating Factor in Children and Adolescents With Acute Myeloid Leukemia: Results of AML-BFM 98. *J Clin Oncol*. 2006;24(27):4499-4506. doi:10.1200/JCO.2006.06.5037

6. Tsukimoto I, Tawa A, Horibe K, et al. Risk-Stratified Therapy and the Intensive Use of Cytarabine Improves the Outcome in Childhood Acute Myeloid Leukemia: The AML99 Trial From the Japanese Childhood AML Cooperative Study Group. *J Clin Oncol*. 2009;27(24):4007-4013. doi:10.1200/JCO.2008.18.7948

7. Rubnitz JE, Inaba H, Dahl G, et al. Minimal residual disease-directed therapy for childhood acute myeloid leukaemia: results of the AML02 multicentre trial. *Lancet Oncol*. 2010;11(6):543-552. doi:10.1016/S1470-2045(10)70090-5

8. Burnett AK, Russell NH, Hills RK, et al. Optimization of Chemotherapy for Younger Patients With Acute Myeloid Leukemia: Results of the Medical Research Council AML15 Trial. *J Clin Oncol*. 2013;31(27):3360-3368. doi:10.1200/JCO.2012.47.4874

9. Pession A, Masetti R, Rizzari C, et al. Results of the AIEOP AML 2002/01 multicenter prospective trial for the treatment of children with acute myeloid leukemia. *Blood*. 2013;122(2):170-178. doi:10.1182/blood-2013-03-491621

10. Cooper TM, Franklin J, Gerbing RB, et al. AAML03P1, a pilot study of the safety of gemtuzumab ozogamicin in combination with chemotherapy for newly diagnosed childhood acute myeloid leukemia: A report from the Children’s Oncology Group. *Cancer*. 2012;118(3):761-769. doi:10.1002/cncr.26190

11. Hasle H, Abrahamsson J, Forestier E, et al. Gemtuzumab ozogamicin as postconsolidation therapy does not prevent relapse in children with AML: results from NOPHO-AML 2004. *Blood*. 2012;120(5):978-984. doi:10.1182/blood-2012-03-416701

12. Creutzig U, Zimmermann M, Bourquin JP, et al. Randomized trial comparing liposomal daunorubicin with idarubicin as induction for pediatric acute myeloid leukemia: results from Study AML-BFM 2004. *Blood*. 2013;122(1):37-43. doi:10.1182/blood-2013-02-484097

13. Gamis AS, Alonzo TA, Meshinchi S, et al. Gemtuzumab Ozogamicin in Children and Adolescents With De Novo Acute Myeloid Leukemia Improves Event-Free Survival by Reducing Relapse Risk: Results From the Randomized Phase III Children’s Oncology Group Trial AAML0531. *J Clin Oncol*. 2014;32(27):3021-3032. doi:10.1200/JCO.2014.55.3628

14. Rubnitz JE, Lacayo NJ, Inaba H, et al. Clofarabine Can Replace Anthracyclines and Etoposide in Remission Induction Therapy for Childhood Acute Myeloid Leukemia: The AML08 Multicenter, Randomized Phase III Trial. *J Clin Oncol*. 2019;37(23):2072-2081. doi:10.1200/JCO.19.00327

15. Aplenc R, Meshinchi S, Sung L, et al. Bortezomib with standard chemotherapy for children with acute myeloid leukemia does not improve treatment outcomes: a report from the Children’s Oncology Group. *Haematologica*. 2020;105(7):1879-1886. doi:10.3324/haematol.2019.220962
