# Supplementary material for: Two-drug versus three-drug induction chemotherapy in pediatric acute myeloid leukemia: a randomized controlled trial
Source: Blood Cancer J. 2022 Sep 6;12(9):131. doi: 10.1038/s41408-022-00726-1 (PMC9444698; doi:10.1038/s41408-022-00726-1)
Supplement: Supplementary file 2 — Supplementary Figure 1 [file 41408_2022_726_MOESM2_ESM.pptx]

## Slide 1
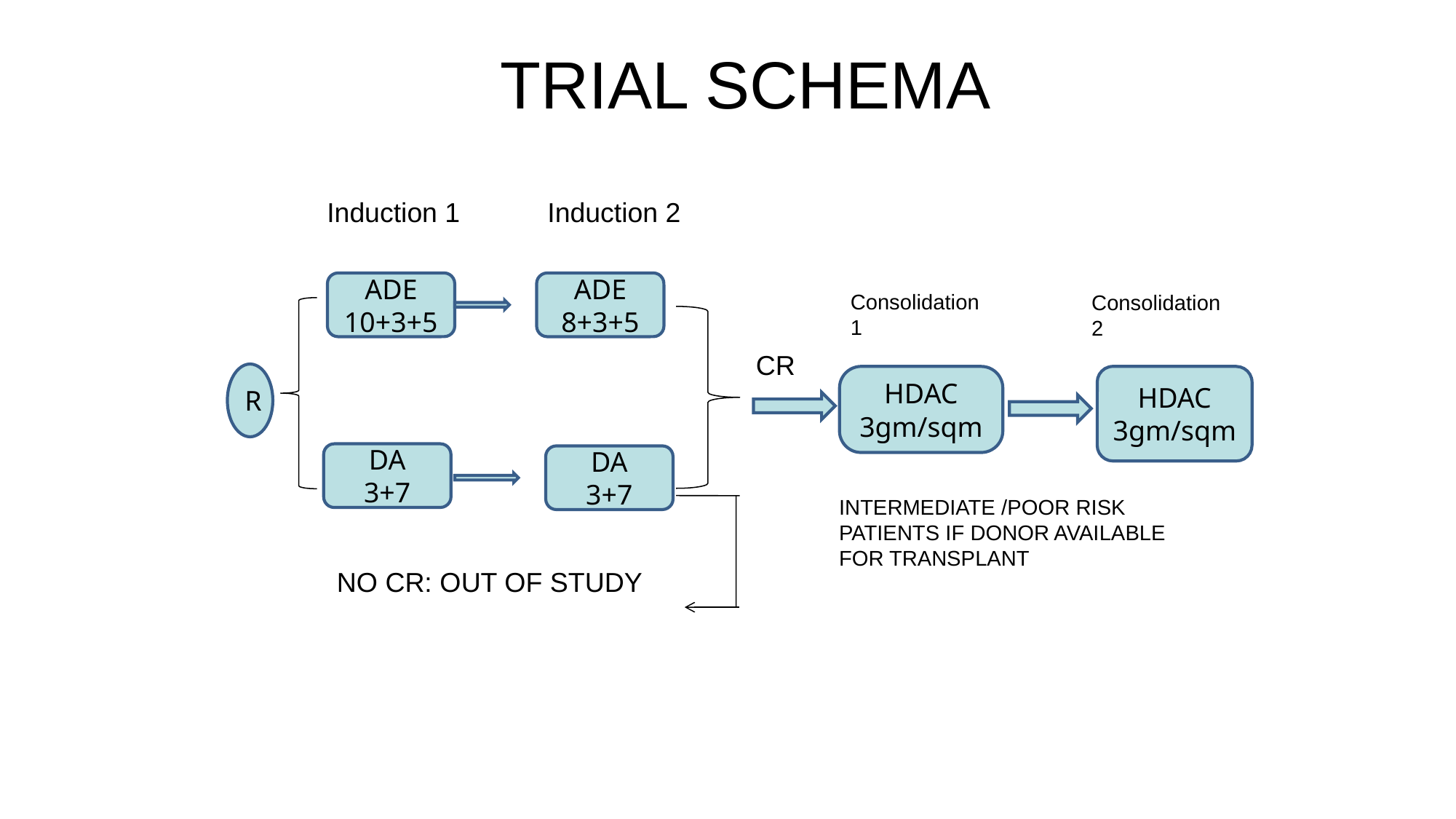

# TRIAL SCHEMA
 Induction 1	Induction 2
				 CR
	 NO CR: OUT OF STUDY
ADE
10+3+5
ADE
8+3+5
Consolidation 1
Consolidation 2
R
HDAC
3gm/sqm
HDAC
3gm/sqm
DA
3+7
DA
3+7
INTERMEDIATE /POOR RISK PATIENTS IF DONOR AVAILABLE FOR TRANSPLANT
