# Supplementary figures and images for: Two-drug versus three-drug induction chemotherapy in pediatric acute myeloid leukemia: a randomized controlled trial
Source: Blood Cancer J. 2022 Sep 6;12(9):131. doi: 10.1038/s41408-022-00726-1 (PMC9444698; doi:10.1038/s41408-022-00726-1)

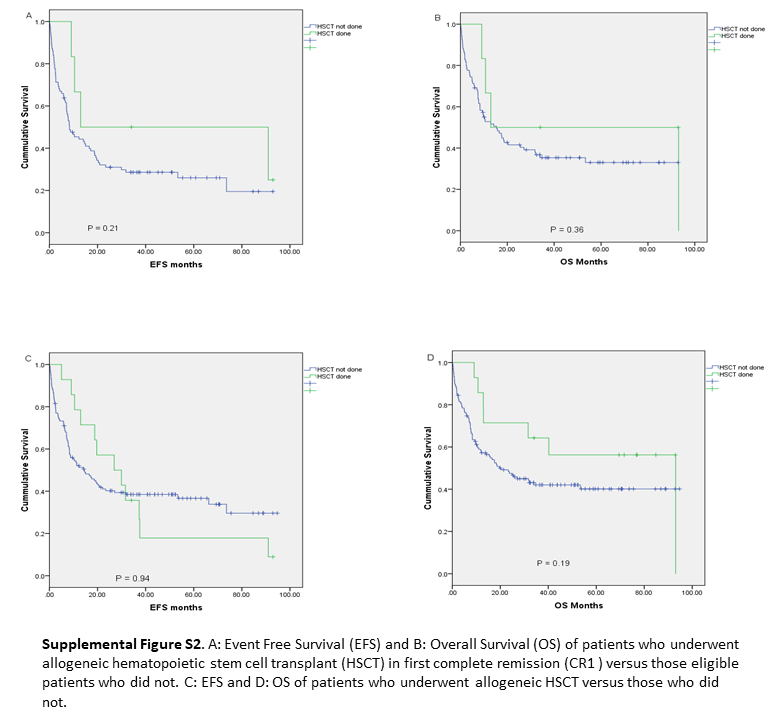

Supplement: Supplementary file 3 — Supplementary Figure 2 [file 41408_2022_726_MOESM3_ESM.tif]
